# Prevalence of *Pentatrichomonas hominis* and *Tritrichomonas foetus* in dogs and cats in Nanchang City, China

**DOI:** 10.1051/parasite/2025016

**Published:** 2025-04-09

**Authors:** Xin-Cheng Jiang, Tao Xiao, Lin-Feng Liu, Ying-Rui Ma, Shu-Ting Xiao, Jia-Jia Shi, Yang Zou, Xiao-Qing Chen

**Affiliations:** 1 College of Animal Science and Technology, Jiangxi Agricultural University, Nanchang Jiangxi Province 330045 PR China; 2 State Key Laboratory for Animal Disease Control and Prevention, College of Veterinary Medicine, Lanzhou University, Lanzhou Veterinary Research Institute, Chinese Academy of Agricultural Sciences, Lanzhou Gansu Province 730046 PR China; 3 College of Animal Science, Jiangxi Agricultural Engineering Vocational College Ganzhou Jiangxi Province 331200 PR China

**Keywords:** *Tritrichomonas foetus*, *Pentatrichomonas hominis*, Dogs and cats, Prevalence

## Abstract

*Tritrichomonas foetus* and *Pentatrichomonas hominis* are two causative agents of trichomoniasis in dogs and cats, manifesting primarily through diarrhea symptoms. However, information on the prevalence and identification of *T. foetus* and *P. hominis* in dogs and cats in China is limited. Thus, to investigate the prevalence of trichomoniasis in dogs and cats in Nanchang city, South China, a total of 405 fecal samples were collected from 111 cats and 294 dogs. The presence of *T. foetus* and *P. hominis* were determined using the nested polymerase chain reaction (PCR) method, targeting the ITS1-5.8SrRNA-ITS2 of *T. foetus*, and 18SrRNA of *P. hominis*. The overall prevalence of *T. foetus* was 15.3% (62/405), with a prevalence of 5.8% (17/294) in dogs and 40.5% (45/111) in cats. The total prevalence of *P. hominis* was 17.3% (70/405), with a prevalence of 22.4% (66/294) in dogs and 3.6% (4/111) in cats. Statistical analysis revealed significant correlations between the prevalence of *T. foetus* and factors including breed, season and environmental conditions in dogs; in cats, there was a significant correlation with season, breeds and age. For *P. hominis*, the different sampling sites of dogs showed a significant correlation. Our results reveal that *T. foetus* is predominantly found in cats and *P. hominis* is predominantly found in dogs in Nanchang city. These findings contributed to effective prevention and control of trichomoniasis in dogs and cats in this region.

## Abstract (fr)

*Tritrichomonas foetus* et *Pentatrichomonas hominis* sont deux agents responsables de la trichomonase chez les chiens et les chats, se manifestant principalement par des symptômes diarrhéiques. Cependant, les informations sur la prévalence et l’identification de *T. foetus* et de *P. hominis* chez les chiens et les chats en Chine sont limitées. Ainsi, pour étudier la prévalence de la trichomonase chez les chiens et les chats de la ville de Nanchang, dans le sud de la Chine, 405 échantillons fécaux ont été prélevés chez 111 chats et 294 chiens. La présence de *T. foetus* et de *P. hominis* a été déterminée par réaction en chaîne par polymérase imbriquée (PCR), ciblant l’ITS1-5.8SrRNA-ITS2 de *T. foetus* et l’18SrRNA de *P. hominis*. La prévalence globale de *T. foetus* était de 15,3 % (62/405), avec une prévalence de 5,8 % (17/294) chez les chiens et de 40,5 % (45/111) chez les chats. La prévalence totale de *P. hominis* était de 17,3 % (70/405), avec une prévalence de 22,4 % (66/294) chez les chiens et de 3,6 % (4/111) chez les chats. L’analyse statistique a révélé des corrélations significatives entre la prévalence de *T. foetus* et des facteurs tels que la race, la saison et les conditions environnementales chez les chiens; chez les chats, il y avait une corrélation significative avec la saison, la race et l’âge. Pour *P. hominis*, il y avait une corrélation significative avec les sites d’échantillonnage. Nos résultats révèlent que *T. foetus* est principalement présent chez les chats et *P. hominis* principalement chez les chiens dans la ville de Nanchang. Ces résultats contribuent à une prévention et un contrôle efficaces de la trichomonase chez les chiens et les chats dans cette région.

## Introduction

Trichomoniasis is a widespread parasitic disease affecting various animals and humans. In dogs and cats, *Tritrichomonas foetus* and *Pentatrichomonas hominis* are two *trichomonad* species, both classified within the *Trichomonadidae* family [13, 21, 36]. *Tritrichomonas foetus* is a dangerous pathogen to many kinds of animal host, including cattle, dogs, cats and pigs. Literature reports have indicated that *T. foetus* might have the capability to infect humans, posing significant potential for zoonotic disease [28]. It has been reported that *T. foetus* is an important cause of chronic and stubborn diarrhea in dogs and cats, parasitizing in the intestines [11, 18]. *Pentatrichomonas hominis* is commonly recognized as a human parasite that parasitizes the intestines of immunocompromized patients and mammals, causing diarrhea [39]. This species exhibits a broad host range including humans, dogs, goats, pigs, monkeys, cattle, cats and farmed wildlife [25, 30]. Immunocompromized patients infected with *P. hominis* typically exhibit symptoms including diarrhea, fever, nausea and vomiting, abdominal pain, bloating and appetite loss [6, 30]. Both *T. foetus* and *P. hominis* exhibit a single form, the trophozoite, throughout the entire life cycle. Both can be transmitted via the fecal-oral route, while *T. foetus* can also be sexually transmitted. In addition, *T. foetus* can parasitize the tissues of the reproductive tract, leading to infertility and abortion occasionally [13, 35].

Nowadays, companion animals are assuming an ever-growing significance in human lives. However, trichomoniasis in dogs and cats can lead to economic losses and pose potential zoonotic risks to humans. Currently, there remains a limited number of studies on trichomoniasis in dogs and cats, especially *P. hominis*. Traditionally, the detection of trichomonad infection relies on the fecal floating method, such as microscopic examinations, isolation and cultivation of insect strains, and electron microscopy. In fact, these methods were unable to accurately differentiate between *P. hominis* with *T. foetus*. Polymerase chain reaction (PCR) has been widely used to identify *Trichomonas* species by amplifying specific genes [29, 34]. Thus, the present study aimed to determine the prevalence and evaluate the risk factors associated with *trichomonad* infection in dogs and cats in Nanchang city, Jiangxi province, China. The findings provide new insights into the epidemiology of *trichomonads* in dogs and cats in south China.

## Materials and methods

### Ethics statement

This experiment was conducted in strict accordance with the experimental animal regulations of Jiangxi Agricultural University. All specimens were collected by anal swab with the consent of the pet owner, and the whole sampling process did not cause damage to animals.

### Specimen collection

**Figure 1:**
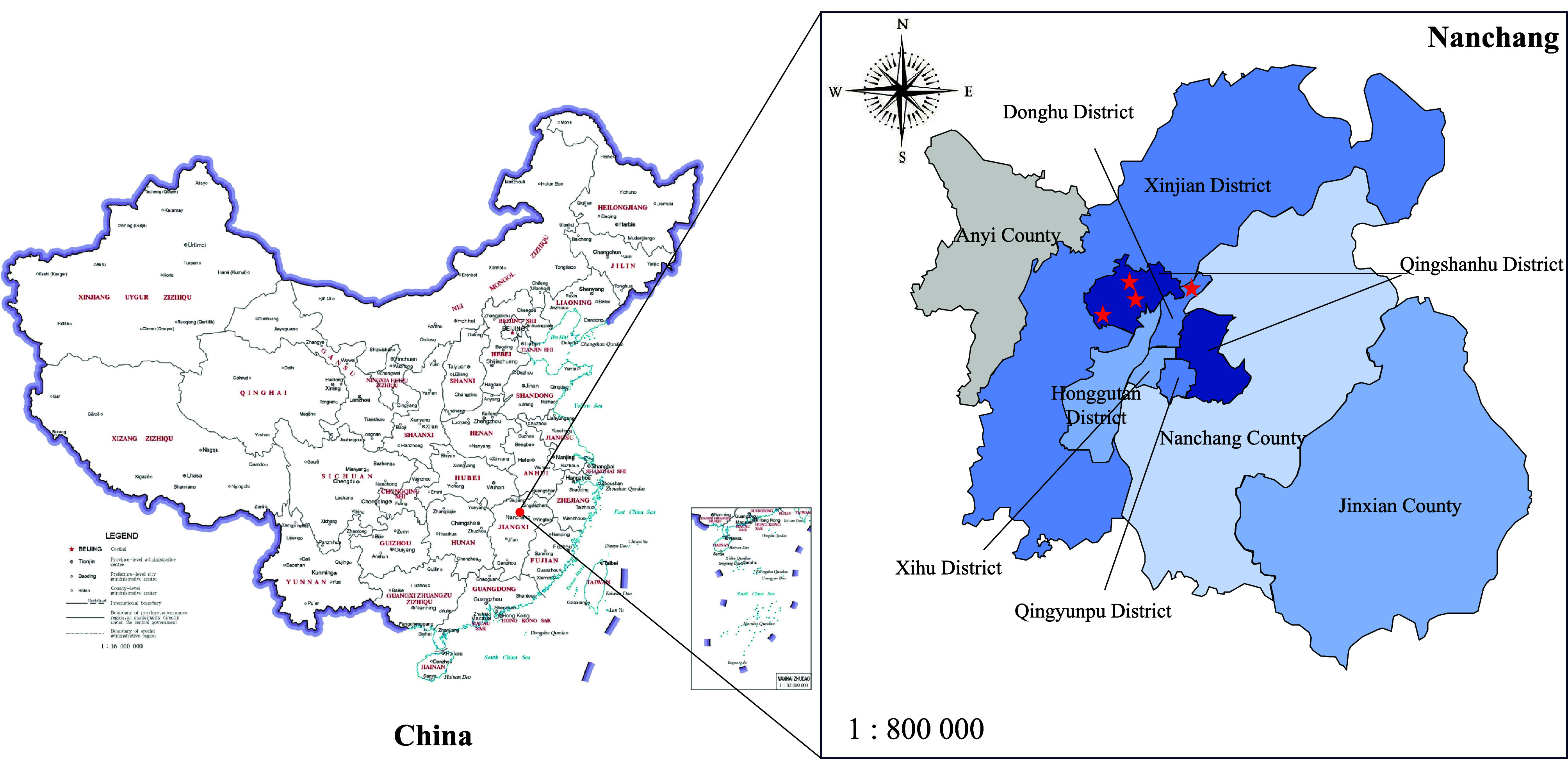
Location of sampling sites.

A total of 405 stool samples, including 111 from cats and 286 from dogs were collected from a pet hospital, a police dog base, and the training base of Jiangxi Agricultural University and a stray dog shelter in the Economic Development Zone of Nanchang City between 2020 and 2023 (Fig. 1). Each fecal sample was collected into a 2 mL stool sampling pipe and labeled with breed, age, gender, collecting location and sampling time. All samples were suitably placed in a sampling box filled with ice packs, and finally stored at −80 °C until DNA extraction.

### Microscopic examination

Each fecal sample was subjected to stool smear preparation, followed by staining using the Giemsa method. The stained smears were then examined under an optical microscope to detect the presence of *Trichomonas* and to observe its structural details.

### DNA extraction

Each sample was cleaned with distilled water by centrifuging at 13,000 rpm for 5 min to remove redundant impurities before DNA extraction. Then, stool samples were extracted using an E.Z.N.A.^®^ Stool DNA Kit (Omega Bio-Tek Inc, Norcross, GA, USA) and the extracted fecal DNA samples were divided and stored at −20 °C until PCR amplification.

### PCR amplification

According to the research conducted by Felleisen *et al.* [10], Gookin *et al.* [13], and Li *et al.* [21], nested PCR primers were synthesized by Tsingke Biotechnology Co., Ltd. For *P. hominis*, the primers OF (ATG GCG AGT GGT GGA ATA) and OR (CCC AAC TAC GCT AAG GAT T) were used for the first round of PCR amplification, while the primers IF (TGT AAA CGA TGC CGA CAG AG) and IR (CAA CAC TGA AGC CAA TGC GAG C) were used for the second round. These primers were designed based on the 18S rRNA gene. Additionally, based on the sequence of ITS1-5.8SrRNA-ITS2 of *T. foetus*, the primers TF-F (CGT ATC AAG CAG GAG GAA GAG GG), TF-R (ATG CTT CAG TTC AGC GGG TCT TC), TF-R4 (CCT GCC GTT GGA TCA GTT TCG TTA A) and TF-R3 (CGG GTC TTC CTA TAT GAG ACA GAA CC) were designed for the nested PCR of *T. foetus.* The first round of the PCR reaction included genomic DNA (2 μL), 10× PCR buffer (Mg^2+^ plus) (2.5 μL), dNTP mixture (0.2 mM), primers (each primer 0.4 μM), Taq DNA polymerase (1.25 U), made up to 25 μL with double distilled water. The second round of PCR system was 25 μL including 17.3 μL sterilized double distilled water, 2.5 μL 10× PCR buffer (Mg^2+^ plus), 2 μL dNTP mixture (2.5 mmol/L), 1 μL upstream primer, 1 μL downstream primer, 0.2 μL Tag DNA polymerase (5 U/L), and 1 μL DNA template (first round amplification product). Annealing temperatures for the nested PCR of *P. hominis* were 59 °C and 61 °C, respectively. Annealing temperatures for *T. foetus* were 57 °C and 54 °C. Each reaction included a positive rate (DNA from *T. foetus* and *P. hominis*) and negative control (double distilled water). The second PCR products were examined by 1.5% (w/v) agarose gel electrophoresis and stained with Gelbule. PCR products of the right size were sequenced.

### Sequence and phylogenetic analyses

Each of the positive PCR products were sent for bi-directional sequencing by Tsingke Biotechnology Co., Ltd. All obtained sequences were subjected to BLAST analysis in NCBI. This allowed for the determination of whether the specimens in this experiment were infected with *P. hominis* or *T. foetus*. Phylogenetic analysis was conducted based on the 18SrRNA gene of *P. hominis* and part of the ITS1-5.8SrRNA-ITS2 gene of *T. foetus*. Using the evolutionary tree drawing software MEGA11 and by the neighbor-joining method (NJ), with the bootstrap parameter set to 1,000, the branches having bootstrap values blow 50 were not viewed in evolutionary tree.

### Statistical analysis

SPSS version 25.0 (IBM SPSS Inc., Chicago, IL, USA) was used to analyze the relationships between *Trichomonas* (*T. foetus* and *P. hominis*) prevalence and independent factors (breeds, gender, age and season) by the chi-square (χ^2^) test. It was considered that a difference in prevalence was significantly related to a factor when the *p*-value was less than 0.05. The infectious risk of *T. foetus* and *P. hominis* was also assessed in dogs and cats, considering various factors. The accuracy of the results was evaluated using odds ratios (ORs) and 95% confidence intervals (CIs).

### Nucleotide sequence accession numbers

The representative nucleotide sequences were submitted to the GenBank database under accession numbers: PP930991–PP930994, PP932478–PP932483 and PP937742–PP937752.

## Results

### Microscopic Examination of *T. foetus* and *P. hominis*

**Figure 2:**
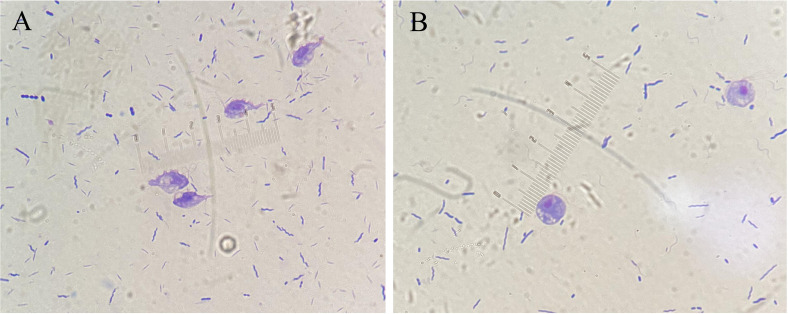
*Trichomonas foetus* detected by light microscopy, Gram staining. A, in a cat fecal sample; B, in a dog fecal sample.

In this study, a total of 294 fecal samples from dogs and 111 fecal samples from cats were stained and observed under a clinical light microscope, using the Giemsa staining method. The results showed that the positive rate of trichomoniasis was 10.20% (30/294) in dogs and 18.9% (21/111) in cats. The parasite was observed and mainly melon-shaped or oval and its size was similar to *Trichomonas.* There were five flagella in the front and back parts of the parasite, which were connected to the fluctuating membrane on the side of *Trichomonas*, and the length of flagella was approximately the same as the body (Figs. 2A and 2B). However, the morphologies under light microscopy did not allow us to differentiate the two species, *T. foetus* and *P. hominis,* effectively.

### Prevalence of *T. foetus* and *P. hominis*

After sequencing identification, 17 dog fecal samples and 45 cat fecal samples tested for *T. foetus* were positive, with an overall prevalence of 15.3% (62/405), resulting in a prevalence of 5.78% (17/294) in dogs and 40.5% (45/111) in cats. For *P. hominis*, 66 dog samples and 4 cat samples were positive, with an overall prevalence of 17.3% (70/405), with a prevalence of 22.5% (66/294) in dogs and 3.6% (4/111) in cats. Statistical analysis showed that the prevalence of *T. foetus* in dogs was significantly correlated with breeds (*p* < 0.01), seasons (*p* < 0.01) and the environment (*p* < 0.01). In cats, the prevalence was significantly correlated with both season and age (*p* < 0.05) (Table 2). For *P. hominis*, the prevalence in dogs was significantly correlated with breeds and environments (*p* < 0.05) (Table 3). It is important to note that due to missing data for some samples, including most of the information on stray dogs and some details regarding the diarrhea status of both dogs and cats, these samples were not included in the calculations.

**Table 1:** Sample information.

Sampling site	Species	Amount
Pet hospital	Dog	131
	Cat	111
Police dog base	Dog	83
Street dog	Dog	80
Total	Dog and Cat	405

**Table 2:** Distribution of *T. foetus* and *P. hominis* of dogs.

Factor		*T. foetus*			*P. hominis*			
		No. Tested	No. positive (%) [95%CI]	OR (95%Cl)	*p*-value	No. positive (%) [95%CI]	OR (95%Cl)	*p*-value
Sex	Male	114	7 (6.14) [1.73–10.55]	1	0.60	24 (21.05) [13.57–28.54]	1	0.73
	Female	100	8 (8.00) [2.68–13.32]	1.33 (0.46–3.81)		23 (23.00) [14.75–31.25]	1.12 (0.59–2.14)	
Age	≤1 years	115	7 (6.09) [1.72–10.46]	1	0.57	29 (25.22) [17.28–33.15]	1.52 (0.78–2.94)	0.22
	>1 years	99	8 (8.08) [2.71–13.45]	1.35 (0.47–3.88)		18 (18.18) [10.58–25.78]	1	
Breed	Crossbred	49	8 (16.33) [5.98–26.68]	4.40 (1.51–12.85)	< 0.01	5 (10.20) [1.73–18.68]	1	0.24
	Purebred	165	7 (4.24) [1.17–7.32]	1		42 (25.45) [18.81–32.10]	3.01 (1.12–8.08)	
Diarrhea situation	Signs	47	7 (14.89) [4.72–25.07]	2.69 (0.92–7.89)	0.06	13 (27.66) [14.87–40.45]	1.41 (0.66–3.02)	0.38
	No signs	131	8 (6.11) [2.01–10.21]	1		28 (21.37) [14.35–28.39]	1	
Season	Spring	7	0 (0.00)	–	< 0.01	2 (28.57) [0–62.04]	2.00 (0.33–12.18）	0.74
	Summer	48	9 (18.75) [7.71–29.79]	13.27 (2.75–64.08)		8 (16.67) [6.12–27.21]	1	
	Autumn	117	2 (1.71) [0.00–4.06]	1		28 (23.93) [16.20–31.66]	1.57 (0.66–3.75)	
	Winter	42	4 (9.52) [0.65–18.40]	6.05 (1.07–34.37)		9 (21.43) [9.02–33.84]	1.36 (0.47–3.93)	
Sampling site	Pet hospital	131	15 (11.45) [6.00–16.90]	5.04 (1.12–22.67)	< 0.01	21 (16.03) [9.75–22.31]	1	0.03
	Police dog base	83	0 (0.00)	–		26(31.33) [21.35–41.30]	2.39 (1.24–4.61)	
	Street dog	80	2 (2.50) [0.00–5.92]	1		19 (23.75) [14.42–33.08]	1.63 (0.81–3.27)	

**Table 3:** Distribution of *T. foetus* and *P. hominis* in cats.

Factor		*T. foetus*			*P. hominis*			
		No. tested	No. positive (%) [95% CI]	OR (95%Cl)	*p*-value	No. positive (%) [95%CI]	OR (95%Cl)	*p*-value
Sex	Male	50	21 (42.00) [28.32–55.68]	1.12 (0.52–2.40)	0.78	2 (4.00) [0.00–9.43]	1.23 (0.17–9.05)	0.84
	Female	61	24 (39.34) [27.09–51.60]	1		2 (3.28) [0.00–7.75]	1	
Age	≤1 years	91	42 (46.15) [35.91–56.40]	4.86 (1.33–17.73)	< 0.05	4 (4.40) [0.18–8.61]	–	0.34
	>1 years	20	3 (15.00) [0.00–30.65]	1		0 (0.00)	–	
Breed	Crossbred	37	10 (27.03) [12.72–41.34]	1	< 0.05	1 (2.70) [0.00–7.93]	1	0.72
	Purebred	74	35 (47.30) [35.92–58.67]	2.42 (1.03–5.71)		3 (4.05) [0.00–8.55]	1.52 (0.15–15.15)	
Diarrhea situation	Signs	31	16 (51.61) [34.02–69.20]	2.24 (0.93–5.42)	0.07	0 (0.00)	–	0.21
	No signs	62	20 (32.26) [20.62–43.89]	1		3 (4.84) [0.00–10.18]	–	
Season	Spring	20	11 (55.00) [33.20–76.80]	11.61 (2.12–63.73)	< 0.05	1 (5.00) [0.00–14.55]	1.02 (0.10–10.37)	0.67
	Summer	21	2 (9.52) [0.00–22.08]	1		0 (0.00)	–	
	Autumn	9	4 (44.44) [11.98–76.91]	7.60 (1.07–54.09)		0 (0.00)	–	
	Winter	61	28 (45.90) [33.40–58.41]	8.06 (1.73–37.66)		3 (4.92) [0.00–10.34]	1	

### Phylogenetic Analysis of *T. foetus* and *P. hominis*

**Figure 3:**
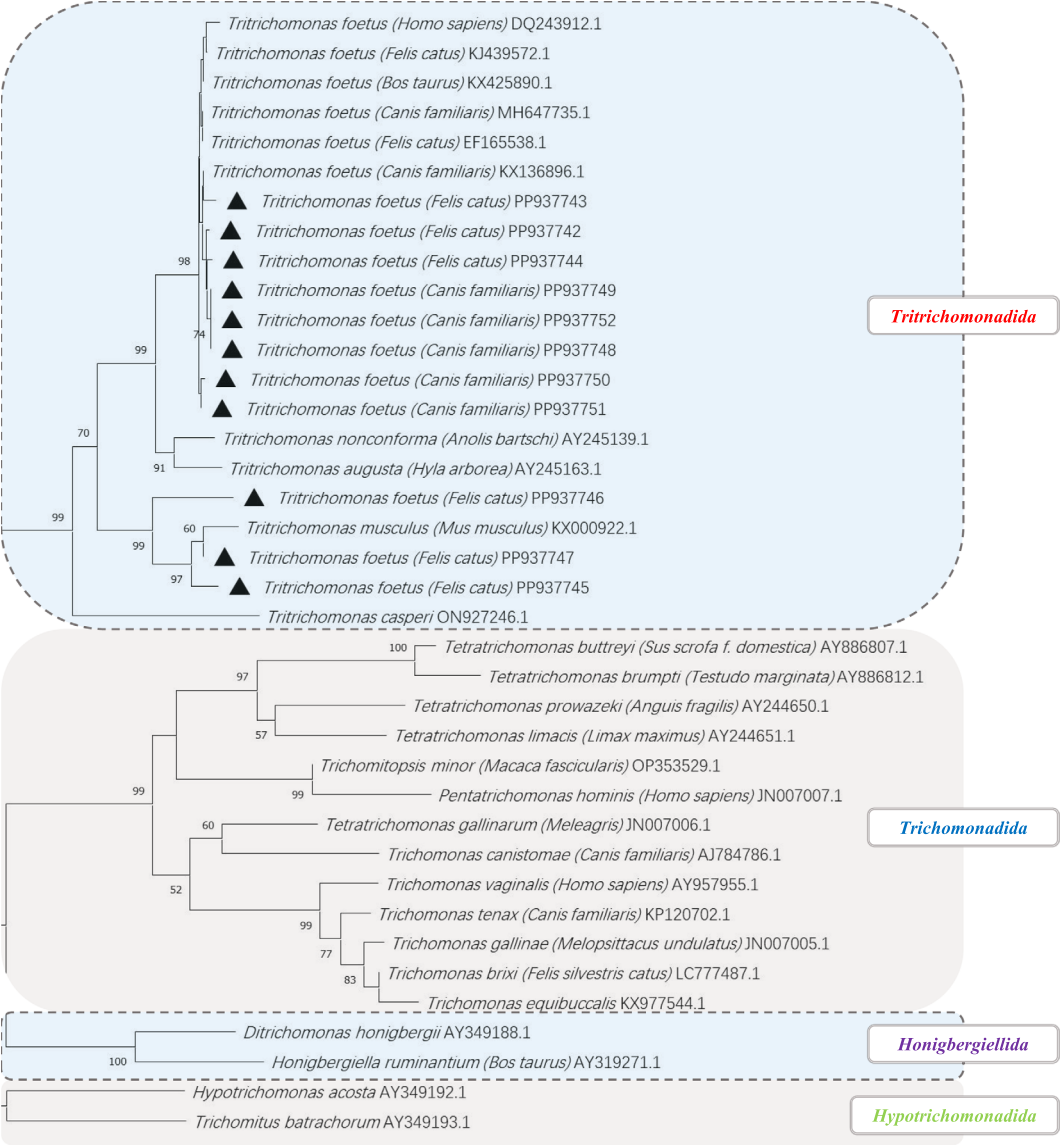
Phylogenetic analysis of *Trichomonas foetus* using the neighbor-joining method (NJ) based on the ITS1-5.8SrRNA-ITS2 gene. The numbers on the branches represent percent bootstrapping values from 1,000 replicates, with values >50% shown in the tree. Solid black triangles: species/subtypes identified in this study.

**Figure 4:**
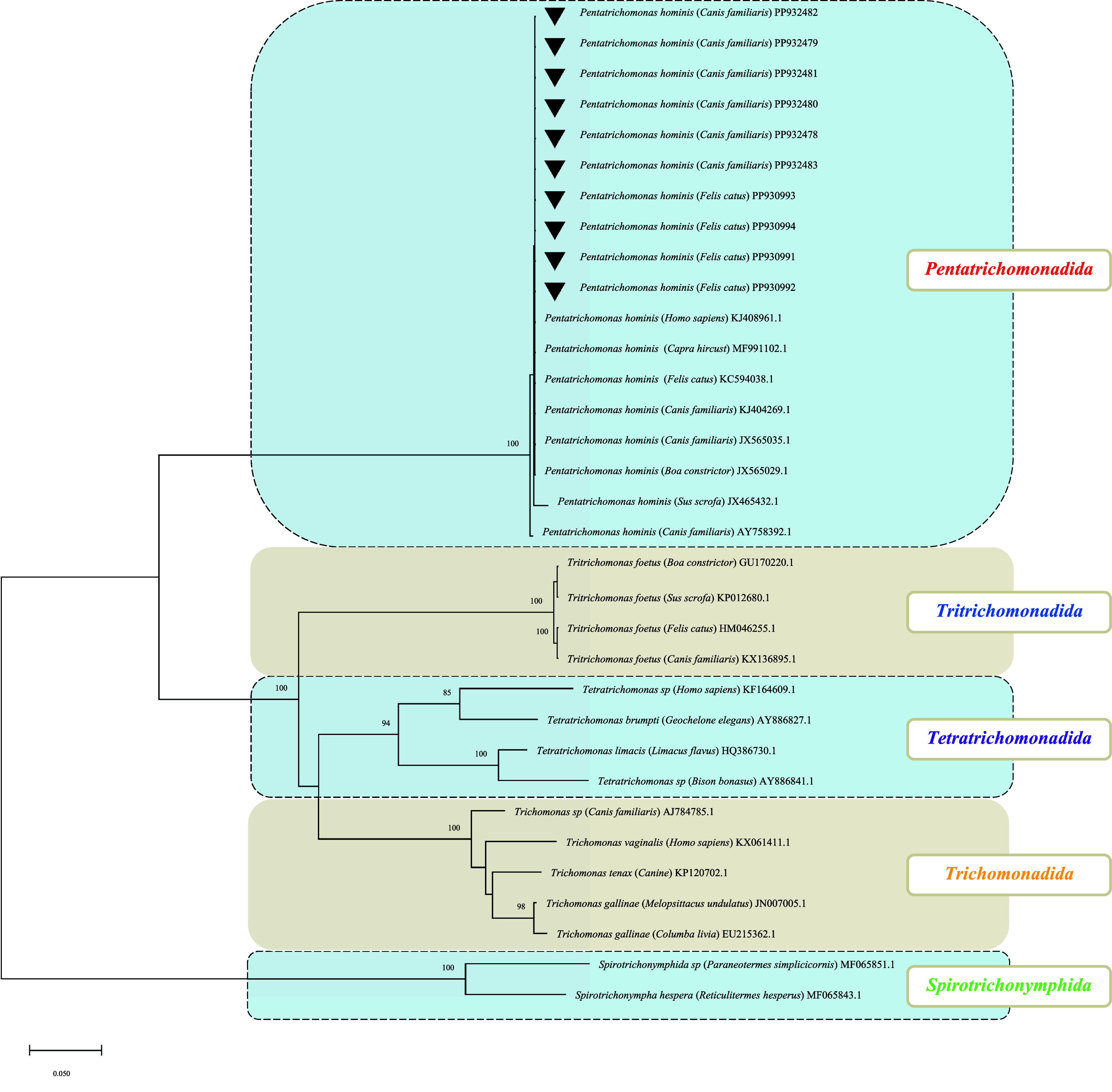
Phylogenetic analysis of *Pentatrichomonas hominis* using the neighbor-joining method (NJ) based on the 18SrRNA gene. The numbers on the branches represent percent bootstrapping values from 1,000 replicates, with values >50% shown in the tree. Solid black triangles: species/subtypes identified in this study.

Phylogenetic analysis shows that the sequences of *T. foetus* in this research clustered into a large branch with isolates of *T. foetus* obtained from other animals with higher bootstrap values (Fig. 3). In the large branch, it is worth noting that three feline *T. foetus* isolates clustered on a small branch, while the other three feline *T. foetus* isolates and five canine *T. foetus* isolates clustered on a branch, indicating differences and mutual transmission of *T. foetus* between dogs and cats. In this study, *P. hominis* sequence all clustered into a large branch obtained from other animals, indicating that *P. hominis* for different animals had a closer relationship (Fig. 4). Meanwhile, Feline *P. hominis* and Canine *P. hominis* are mixed together, indicating the mutual transmission of *P. hominis* in dogs and cats, and even among other animals.

## Discussion

This study was the first examination of the prevalence of *T. foetus* and *P. hominis* in dogs and cats in Nanchang city, China. We found that the detection rate of microscopic examination was low, making it impossible to differentiate between *T. foetus* and *P. hominis*. Based on PCR, we found that the overall prevalence of *T. foetus* in dogs and cats in Jiangxi Province was 5.8% (17/294) and 40.5% (45/111), respectively. The results were in alignment with the current prevalent trend of *T. foetus* in dogs and cats in China, for which the prevalence in dogs ranged from 0.64% to 54.7%, and in cats ranged from 10.1% to 47.4% [17, 22, 24]. In other research, the prevalence of *T. foetus* in dogs was only 5.3% (2/38) in the United States [36], the prevalence of *T. foetus* in cats was 6.7% in South Korea [33], 1.39% (3/215) in Thailand [20], and 20.52% (24/117) in Poland [5], which is consistent with our study. Here, we found that the prevalence of canine *T. foetus* infection was higher in non-breed dogs (16.3%, 8/46) than purebred dogs (4.2%, 7/165). Nevertheless, no pertinent research exists to corroborate our findings. There is still a lack of evidence regarding whether breeds are predisposed to parasite infection at the genetic or immunological level. Our findings also indicated that the prevalence of canine *T. foetus* was related to season, for which the positive rate of canine *T. foetus* infection was higher in summer than in autumn and winter. The warm conditions of spring and summer enable parasites to thrive for an extended duration within moist feces [14], which may be conducive to spreading. Interestingly, we discovered that the living condition was also an important factor that influenced the prevalence of *T. foetus*, for which the positive rate in dogs in pet hospitals (11.5%, 15/131) was higher than at the police dog base (2.5%, 2/80). In this study, the findings regarding potential risk factors for canine *T. foetus* infection indicated that age, sex, and diarrhea status exhibited no significant differences, aligning closely with the observations reported in previous studies [12, 24].

Our results indicated a significant correlation between the young age of cats (<1 year old) and *T. foetus* infection, which was consistent with reports in Spain [31], the United Kingdom [16], Greece [37] and China [38]. Our results confirmed that the positive rate of feline *T. foetus* infection was higher in purebred cats (47.3%, 35/74) than non-purebred cats (27.0%, 10/37), for which the result was reversed for dogs and similar to the findings of other authors [36]. The elevated incidence of tritrichomonosis in purebred cats, predominantly residing in multi-cat households, may be caused by high density. This relationship heightens the risk of introducing parasites into cat houses and facilitates its further transmission [2]. Similarly, our investigation revealed that the prevalence of feline *T. foetus* infection was correlated with season. It was notable that the infection rates in spring, autumn and winter showed no significant differences, whereas in summer, the rate was the lowest. However, some reports found that the infection rate of feline *T. foetus* was higher in spring and summer than in winter. This might result from the uneven seasonal distribution of the samples in this study. Therefore, the association between the susceptibility of *T. foetus* and seasons is worthy of further research. Some studies have found that the susceptibility of feline *T. foetus* was not gender-biased [1, 3], and our results confirmed this. Diarrhea is a major clinical manifestation of feline *T. foetus* [1, 17]. Some studies have reported a significant correlation between diarrhea symptoms and feline *T. foetus* infection [7, 15], and there were also reports that indicated no significant difference between feline *T. foetus* infection and fecal status [5, 19]. In this study, there was also no significant correlation between diarrhea symptoms and feline *T. foetus* infection, which might be influenced by the sample size.

In this study, the prevalence of *P. hominis* in dogs and cats in Jiangxi province was 22.4% (66/294) and 3.6% (4/111), which was similar to the epidemic trend of canine *P. hominis* in the northern (27.4%, 69/252) and eastern (31.4%, 99/315) regions of China, and feline *P. hominis* infection was also consistent with previous research in China (5.3% 3/57) [5, 23]. The positive rate of *P. hominis* in dogs was 47.4% (18/38) in the United States, 15.8% (34/215) in France and 6.9% (38/544) in Japan; the prevalence of *P. hominis* in cats was 1.9% (2/103) in the United States, 17.7% (21/119) in Thailand and 0.5% (2/409) in Japan [26, 32], which is consistent with the findings of our study. Our results showed that the feeding environment had a certain effect on canine *P. hominis* infection, for which the prevalence rates in dogs in police service (31.3%, 26/83) and in street dogs (23.8%, 19/80) were higher than in pet hospital dogs (16.0%, 21/131). The stray dog rescue center and the police dog training facility could serve as gathering points for dogs, potentially facilitating the transmission of canine *P. hominis*. Conversely, the positive rate of canine *P. hominis* infection was relatively low among individual animals receiving treatment at pet hospitals. Our results also indicated that sex, age, breed, diarrhea manifestations and season did not significantly influence the infection rate of canine *P. hominis* in dogs. Meanwhile, no significant differences were found for factors of infection with feline *P. hominis*. Therefore, it remains essential to expand the sample size for further investigation.

Both *T. foetus* and *P. hominis* have an extensive host range [3, 9, 22]. First, *T. foetus* has been isolated from a variety of pets and farm animals, with the same strain known to infect cattle and pigs [27], but different genotypes infect cattle and cats [4, 8]; the origins of dog infections remain unclear. It was reported that more than two *T. foetus* genotypes capable of colonizing had an extensive range of hosts, including humans [9]. Second, *P. hominis* has been isolated from a variety of pets and farm animals [27], but little is known about its infection routes and epidemiology; the same strain could be circulating between all identified hosts. In this study, the phylogenetic analyses demonstrated that the sequences of *P. hominis* and *T. foetus* from the current study consistently cluster with their respective trichomonad counterparts. This clustering pattern suggested the potential for interspecies transmission between cats and dogs. Notably, a subset of feline *T. foetus* sequences had been observed to diverge from the main cluster, clustering closely with *Tritrichomonas musculus*, which is likely to be derived from another species. This divergence indicates that *T. foetus* might involve a risk of cross-species transmission.

## Conclusions

This study investigated the prevalence of *T. foetus* and *P. hominis*, two causative agents of trichomoniasis, in dogs and cats in Nanchang city, south China. The overall prevalence of *T. foetus* was 15.3% (62/405), with a significantly higher prevalence in cats (40.5%, 45/111) compared to dogs (5.8%, 17/294). For *P. hominis*, the overall prevalence was 17.3% (70/405), with dogs having a higher prevalence (22.4%, 66/294) than cats (3.6%, 4/111). Statistical analysis revealed significant correlations between the prevalence of *T. foetus* and factors such as breed, season and environment (*p* < 0.01) in dogs, and with season and age (*p* < 0.05) in cats. Similarly, statistical analysis revealed significant correlations between the prevalence of *P. hominis* and environment (*p* < 0.05) in dogs. No factors were identified as being related to the prevalence of *P. hominis* in cats. The risk of trichomoniasis transmission in dogs and cats is significantly elevated in environments characterized by a high concentration of companion animals, such as breeding facilities and households with multiple companion animals. The phylogenetic analyses indicated that *T. foetus* might involve a risk of cross-species transmission. One of the limitations of this study was the sample size, which might not be sufficient to represent the entire population of dogs and cats in Nanchang City, China. Future studies should expand the sample size and cover more diverse geographical areas to provide a more comprehensive understanding of the pathogens. Moreover, incorporating long-term follow-up data and exploring the impact of various environmental factors on infection rates would enhance the understanding of the epidemiology of *T. foetus* and *P. hominis* in pets.
